# Le diverticule de l'urètre féminin: à propos de 18 cas

**DOI:** 10.11604/pamj.2014.18.78.4573

**Published:** 2014-05-24

**Authors:** Mouad Statoua, Jihad El Ghanmi, Tarik Karmouni, Khalid El Khader, Abdellatif Koutani, Ahmed Iben Attya

**Affiliations:** 1 Clinique Urologique « B », CHU Ibn-Sina, Rabat, Maroc

**Keywords:** Diverticule de l'urètre, femme, urétrocystographie, exérèse transvaginale, Diverticulum of the urethra, woman, urethrocystography, transvaginal excision

## Abstract

Le diverticule de l'urètre ou poche sous urétrale est une affection rare, d’étiopathogénie non clairement établie, le diagnostique est clinique confirmé par l'urétrocystographie et le traitement est principalement chirurgicale consistant en une diverticulectomie par voie transvaginale. Nous rapportons l'expérience de notre service dans la prise en charge de cette affection en présentons une étude rétrospective sur une durée de 14 ans (entre 2000 et 2014) où on a pris en charge 18 patientes qui présentait un diverticule de l'urètre, l’âge moyen était de 36 ans, une symptomatologie urinaire ramenait les patientes à consulter où le diagnostic de DU a été posé par examen clinique confirmé en précisant ses caractéristiques en urétrocystographie, la prise en charge était chirurgicale et consistait en une diverticulectomie par voie transvaginale. Les suites post-opératoire était simples, la sonde vésicale retirée en moyenne 5,8 jours après l'intervention, on n'a noté aucune complication chez toute nos patientes, hormis un cas de récidives repris. Devant des troubles mictionnels récidivants de la femme, il est indispensable de rechercher un diverticule uréthral à l'examen clinique. La diverticulectomie transvaginale est l'intervention de choix offrant les meilleurs résultats.

## Introduction

Le diverticule de l'urètre ou poche sous urétrale est défini comme une hernie de la muqueuse urétrale à travers les fibres musculaires lisses qui compose la paroi urétrale. Il forme un cul de sac communiquant souvent avec la lumière urétrale par un collet et fait protrusion au niveau de la paroi vaginale [1, 2]. Décrit la première fois par HEY en 1786 [3], c'est une pathologie rare dont la physiopathologie est mal élucidée, son diagnostic est essentiellement clinique confirmé par la radiologie et son traitement est chirurgicale. Nous rapportons notre expérience dans la prise en charge de 18 patientes et nous analysons les particularités diagnostiques et aspects thérapeutiques de cette pathologie ainsi que les différentes hypothèses étiopathogéniques.

## Méthodes

**Figure 1:**
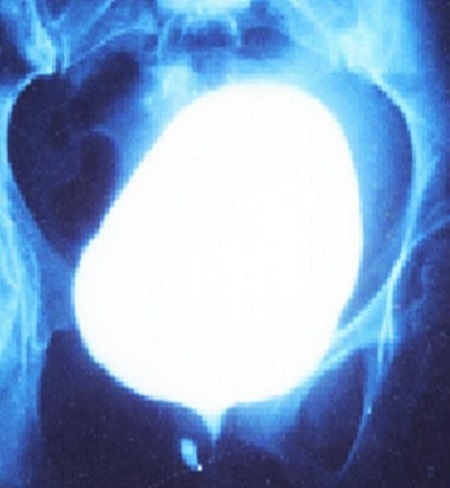
Image d'uréthrocystographie rétrograde et mictionnelle en phase mictionnel montrant un petit diverticule de 6 mm de diamètre

**Figure 2:**
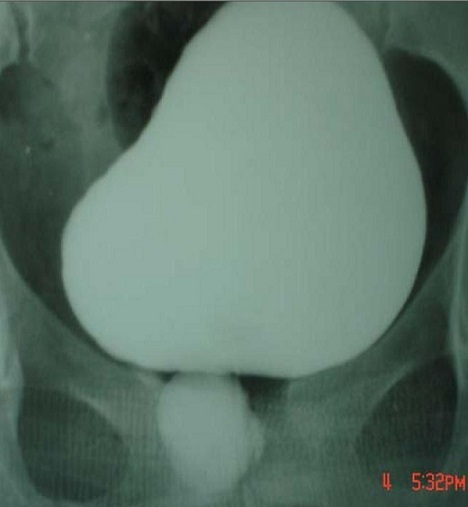
Cliché d'uréthrocystographie rétrograde et mictionnelle montrant une grosse poche sous urétrale

**Figure 3:**
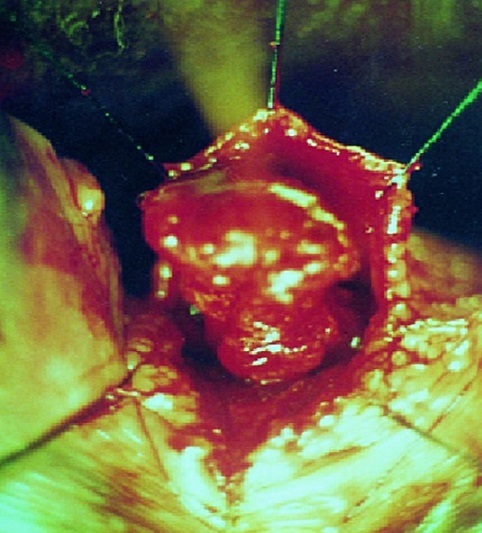
Image per-opératoire d'un diverticule urétrale après sa dissection

De janvier 2000 à janvier 2014, 18 patientes ont été hospitalisées dans notre service pour cure d'un diverticule urétral. L’âge moyen des patientes était de 36ans, le nombre moyen d'accouchements était de 2 par femme dont 8 étaient dystociques. Toutes ces patientes présentaient des signes urinaires du bas appareil: essentiellement des cystites récidivantes. Le délai moyen entre le début des symptômes et le diagnostic était 9 mois (extrêmes: 6-38mois). A l'examen gynécologique, le diverticule était palpable le long du trajet urétral chez 16 patientes. Les deux tiers des diverticules étaient distaux situés à moins de 2cm du méat urétral. L'ECBU était infecté chez 75% des cas, avec une prédominance d'E.coli. L'UCG a permis d'objectiver le diverticule chez la totalité des patientes (Figure 1, Figure 2). Toutes ces patientes ont bénéficié d'une intervention chirurgicale consistant en une exérèse du diverticule (diverticulectomie) par voie vaginale. Installées en position gynécologique après antibioprophylaxie et sous anesthésie loco-régionale (rachianesthésie). Après la pose d'une sonde vésicale, l'abord du diverticule se fait par voie vaginale, l′incision vaginale antérieure était transversale (8 fois), arciforme (5 fois) ou en U inversé (5 fois). La taille moyenne des diverticules était de 19 mm (extrêmes 6-30mm). La clef de la diverticulectomie est la dissection du collet diverticulaire qui sera fermé au vicryl 4/0 en points séparés; à cette fermeture s′associent celles du fascia péri-urétral et de la paroi vaginale antérieure (Figure 3). Toutes les patientes ont gardé le drainage vésical par sonde urétrale pour une durée moyenne de 5,8 jours (extrêmes 5-7 jours).

## Résultats

La durée du séjour moyen était de 6 jours (5 à 8 jours), au retrait de la sonde vésicale aucune patiente n'a rapporté de gène fonctionnelle et un calendrier mictionnel a été réalisé le jour du retrait de la sonde vésicale autorisant la patiente a sortir le lendemain vu que tous les résultats étaient satisfaisants, aucune complication à cours terme n'a été signalé avec un recul moyen de trois ans hormis une récidive qui est survenu chez une patiente 2 ans après son intervention et qui a eu un accouchement dystocique 3 mois avant la récidive, une diverticulectomie de novo fut réalisée chez cette patiente avec bonne évolution. L’étude anatomopathologique de la pièce a objectivé la présence de tissue urotéliale avec une métaplasie malpighienne chez 11 patientes et des signes inflammatoires non spécifiques chez 7 patientes.

## Discussion

Le diverticule de l'urètre qui correspond à une hernie de la muqueuse urétrale à travers un defect musculaire de la paroi urétrale est d’étiopathogénie incertaine vu que plusieurs avancées hypothétiques ont été avancées. Le premier cas de DU de la femme a été rapporté en 1786 par William Hey mais la première excision chirurgicale de ce diverticule n'a été réalisé qu'en 1875 par Tait [1, 3]. Le diagnostic du DIU est habituellement porté entre la troisième et la cinquième décade de la vie [4], de rares cas aient été décrits chez des nouveaux-nés ou chez des jeunes filles. L′âge moyen lors du diagnostic est de 37,5 ans [2]. La pathogénie des diverticules urétraux féminins reste discutée. Plusieurs hypothèses ont été avancées: congénitale, iatrogène, traumatique et infectieuse [5, 6]. En faveur des diverticules congénitaux: la présence des formes néonatales ou découvertes chez la fillette qui se développeraient aux dépens de reliquats embryonnaires (canal de Gartner) [5]. Certains auteurs ont avancé l′hypothèse d′une origine traumatique de ces diverticules [4]. L′hypothèse la plus communément admise est celle de ROUTH [6], selon laquelle l′infection et l′obstruction répétées des glandes péri-urétrales aboutissent à la formation des kystes sous urétraux. Les germes les plus fréquemment en cause sont le gonocoque et l′Eschérichia coli. Ces glandes périurétrales se drainent pour plus de 90% d′entre elles au niveau du tiers moyen ou distal de l′urètre. Le diagnostic est évoqué sur la triade symptomatique des trois «D» selon les anglosaxons: dribbling, dyspareunia, dysuria [7]. L′issue de pus par le méat urétral au cours des manoe'uvres exprimant la poche tels les rapports sexuels et les toilettes vaginales, la miction en 2 temps par vidange de la poche sont très évocateurs du diagnostic. Les infections urinaires à répétition, les signes irritatifs à type de pollakiurie et d′impériosités sont rencontrés une fois sur deux. Cependant 4 à 20% des DU sont asymptomatiques et sont retrouvés fortuitement lors d'un examen clinique ou radiologique réalisé pour autre chose [2].

Par ailleurs, l′examen clinique permet d′établir le diagnostic de DU dans plus de 60% 90% de cas [2, 7] parfois cet examen est strictement normal. Typiquement la poche se présente sous forme d′une tuméfaction vaginale antérieure sensible. La pression de cette masse ferait sourdre, dans la majorité des cas, du pus, de l'urine ou du sang par le méat urétral. Une induration du diverticule peut faire suspecter l'existence d'un calcul, ou une tumeur [1]. En fait, le diagnostic de DU nécessite que l'on y pense systématiquement devant toute femme présentant des troubles mictionnels [1]. La majorité des DU semble situer au niveau de la moitié ou du tiers distal de l'urètre correspondant à la localisation des glandes péri-urétrales. Le diagnostic différentiel se pose avec les autres masses urétrales et intravaginales tels les abcès des glandes périurétrales de Skéne, les kystes du canal de Gartner, le prolapsus d′une urétérocèle et les lésions périméatiques, un fibromyome péri-urétral ou vaginal, un hémangiome, des varices urétrales, une endométriose urétrale ou enfin une métastase vaginale [1]. L′urétrocyctoscopie, après vérification de la stérilité des urines, peut aider à localiser le collet diverticulaire qui est le plus souvent sur la paroi postérieure entre 4 et 8 heures [1]. L'uréthrocystographie rétrograde et mictionnelle (UCRM) est l'examen radiologique de première intention réaliser pour mettre en évidence le DU [2], le cliché mictionnel permet d'objectiver le collet diverticulaire et apprécier les caractéristiques du diverticule [1]. la littérature récente fait état de nouvelles modalités diagnostiques, comme l'imagerie par résonance magnétique nucléaire (IRM) avec antenne endorectale [8]; l′échographie endo-vaginale [5] endorectale [9] translabiale [10] et endo-urétrale [8], L′IRM semble améliorer la détection de ces diverticules, notamment les petits diverticules et les diverticules non-communiquants, l’échographie par voie vaginale ou endourétrale est un examen non invasif dont la fiabilité est comparable à celle de l'urétrocystographie [5]. Elle peut par ailleurs aider pour faire le diagnostic des diverticules dont le collet est fermé. La diverticulectomie par voie transvaginale constitue le seul traitement curateur. Certains auteurs privilégient l'incision en U inversée qui évite le chevauchement des sutures, et prévient les fistules ainsi que les sténoses postopératoires. La dissection du collet et sa fermeture en 3 plans incluant l'urètre, le fascia péri-urétral et la paroi vaginale, permettent de prévenir les fistules et les récidives [1, 2]. Le drainage vésical est assuré par une sonde urétrale ou une cystostomie pendant une durée de 10 jours.

La chirurgie de ces diverticules expose à des complications de type: fistules, sténose urétrale, incontinence urinaire d'effort ou par instabilité [6]. La fréquence de ces complications est estimée entre 3,3% et 10% dans la littérature[6]. Les récidive sont exceptionnelles dues essentiellement à une infection urétrale active; les réactions inflammatoires locales et les difficultés de drainage vésicale [11]. La meilleure prévention de ces complications reste la maîtrise de la chirurgie vaginale et la prudence lors de la dissection du collet diverticulaire et l'exérèse complète du diverticule [2].

## Conclusion

Le diverticule de l'urètre féminin est une pathologie plus méconnue que rare dont l’étiopathogénie est encore mal cernée. Les signes fonctionnels sont dominés par les troubles mictionnels, les infections urinaires récidivantes et la dyspareunie. Le diagnostic essentiellement clinique est confirmé par l'UCRM, voire par l’échographie transvaginale. La cure d'exérèse chirurgicale par diverticulectomie transvaginale en position dorsale représente l′intervention de référence.
